# Impact of Interferon-α Receptor-1 Promoter Polymorphisms on the Transcriptome of the Hepatitis B Virus-Associated Hepatocellular Carcinoma

**DOI:** 10.3389/fimmu.2018.00777

**Published:** 2018-04-16

**Authors:** Timokratis Karamitros, George Papatheodoridis, Dimitrios Paraskevis, Angelos Hatzakis, Jean L. Mbisa, Urania Georgopoulou, Paul Klenerman, Gkikas Magiorkinis

**Affiliations:** ^1^Department of Zoology, University of Oxford, Oxford, United Kingdom; ^2^Department of Microbiology, Public Health Laboratories, Hellenic Pasteur Institute, Athens, Greece; ^3^Academic Department of Gastroenterology, Laiko General Hospital, Medical School of National and Kapodistrian University of Athens, Athens, Greece; ^4^Department of Hygiene and Epidemiology and Medical Statistics, Medical School of National and Kapodistrian University of Athens, Athens, Greece; ^5^Virus Reference Department, Public Health England, London, United Kingdom; ^6^Department of Microbiology, Molecular Virology Laboratory, Hellenic Pasteur Institute, Athens, Greece; ^7^Peter Medawar Building for Pathogen Research and Translational Gastroenterology Unit, University of Oxford, Oxford, United Kingdom

**Keywords:** hepatitis virus B, interferon-α receptor type-1 promoter, hepatocellular carcinoma, interferon-α receptor, polymorphism, transcriptome, RNAseq, the Cancer Genome Atlas project

## Abstract

**Background and aims:**

Genetic polymorphisms within the promoter of interferon-α receptor type-1 (IFNAR1) have been associated with the susceptibility to and the outcome of chronic hepatitis B virus (HBV) infection. However, the impact of these polymorphisms in the transcriptome of the HBV-associated hepatocellular carcinoma (HCC) remains largely unexplored.

**Methods:**

Using whole-genome and exome sequencing data from The Cancer Genome Atlas project, we characterized three single-nucleotide polymorphisms (SNPs: −568G/C, −408C/T, −3C/T) and one variable number tandem repeat [VNTR: −77(GT)n] within the IFNAR1 promoter sequence in 49 HCC patients. RNAseq data from 10 genotyped HCC samples were grouped according to their −77VNTR or −3SNP genotype to evaluate the impact of these polymorphisms on the differential expression on the HCC transcriptome.

**Results:**

There is a fourfold higher impact of the −77VNTR on the HCC transcriptome compared to the −3SNP (*q* < 0.1, *p* < 0.001). The expression of the primary IFNAR1 transcript is not affected by these polymorphisms but a secondary, HCC-specific transcript is expressed only in homozygous −77VNTR ≤8/≤8(GT)n samples (*p* < 0.05). At the same time, patients carrying at least one −77VNTR >8(GT) allele, presented a strong upregulation of the fibronectin-1 (FN-1) gene, which has been associated with the development of HCC. Gene Ontology and pathway enrichment analysis of the differentially expressed genes revealed a strong disruption of the PI3K–AKT signaling pathway, which can be partially triggered by the extracellular matrix FN-1.

**Conclusion:**

The IFNAR-1 promoter polymorphisms are not involved in the expression levels of the main IFNAR-1 transcript. The −77VNTR has a regulatory role on the expression of a secondary, truncated, HCC-specific transcript, which in turn coincides with disruptions in cancer-associated pathways and in FN-1 expression modifications.

## Introduction

Worldwide, more than two billion people have been infected with hepatitis B virus (HBV) and approximately 250 million individuals are chronically infected (1). Infected patients can be inactive chronic HBV carriers (IC) (eAg-negative, eAb-positive, with low levels of HBV DNA and no evidence of liver inflammation) or present with the progressive chronic hepatitis B (CHB) (2–5). HBV is responsible for >50% of hepatocellular carcinomas (HCCs) worldwide (2, 6, 7). On the other hand, HBeAg-negative ICs have a more benign prognosis with very low risk of cirrhosis or HCC, as indicated by long-term follow-up studies (8–10).

The genetic profile of the patient plays a substantial role in the clinical outcome of HBV infection (11–13). The virus also modulates cellular mechanisms and signal pathways during the course of the infection. For example, the persistent expression of the HBV x antigen (HBxAg) is correlated with the development of fibrosis and cirrhosis during CHB, as it can activate fibronectin-1 (FN-1) gene, through the induction of the nuclear factor kappa B (NF-kappa B or NFkB) (14). FN is an omnipresent extracellular matrix glycoprotein. Plasma FN and cellular FN have distinct properties and roles in the strictly regulated mechanism of tissue repair (15). FN is a very important component of ECM and any dysfunction in the fibrinogenesis mechanisms can lead to the development of fibrotic disease (16).

Interferons (IFN)-α/β are cytokines involved in both innate and adaptive immune responses (17, 18), thus play a pivotal role as cancer defense mechanisms (19). Interferon-induced signal transduction pathways represent a fine-tuned network of interactions, which are triggered upon binding of IFN on its receptor (IFNR) (20–22). IFNs are shown to regulate the transcription levels of more than 2,000 genes, which compose the *Interferome*, a gene-network created by integrating information collected from high-throughput experiments (23). As IFNs are anticancer and antiviral cytokines, it is expected that the genetic profiles of the genes involved in these signal transduction pathways (e.g., IFNR) will have an impact on the susceptibility of the patients to cancer and, when HBV is involved, to CHB and HBV-related HCC.

IFN-α is administrated as part of the first-line therapies for CHB (2, 24). The compatible receptor (IFNAR) consists of two subunits IFNAR-1 and IFNAR-2 (25). A number of polymorphisms in the promoter of IFNAR-1 gene and also in its coding sequence have been described (26–29). Those observed in the promoter—three SNPs and one variable number tandem repeat (VNTR) at positions −568, −408, −3 and −77, respectively—are believed to affect the expression of the receptor and are associated with the clinical outcome of HBV infection (27, 30, 31).

We have previously shown that the same polymorphisms are associated with the clinical phase of HBeAg-negative chronic HBV infection in Caucasians. Briefly, patients with genotypes −568GC/CC, −408CT, and −3CT and patients with less than 8(GT) repeats in the −77VNTR were more frequent among inactive carriers (IC) vs. patients with HBeAg-negative CHB (32). Notably, the (GT)-repeats in the −77VNTR were strongly associated with the clinical outcome of the patients in our study; homozygotes carrying both alleles with ≤8(GT)-repeats, were more likely to be ICs, compared to those carrying both alleles with >8(GT) repeats (OR = 7.14, *p* ≤ 0.001).

In this study, we use whole-genome sequencing (WGS), whole-exome sequencing (WXS), and RNAseq data derived from The Cancer Genome Atlas (TCGA) project in combination with a bioinformatics pipeline, which efficiently and accurately genotypes the −77VNTR. We estimate the distribution of the genetic variations of the four IFNAR-1 promoter polymorphisms and we investigate the impact of −77VNTR and −3SNP polymorphisms on the regulation of HCC transcriptome and interferome. IFNAR-1 is transcribed into three transcripts, ENST00000270139 (which is translated into the receptor), ENST00000493503, and ENST00000442071; performing differential expression analysis in patients grouped according to their −77VNTR genotype, we show that −77VNTR modifies the transcriptional profile of IFNAR-1 gene by controlling the expression of the last one, which comprises only a fibronectin III domain and is translated into a truncated form of the receptor. The expression of this transcript coincides with significantly lower expression of FN-1. Finally, the PI3K–AKT signaling pathway, which is partially triggered by FN-1, was significantly enriched with differentially expressed genes. Our results indicate that this secondary transcript could potentially act as a regulatory element, but its functional role remains largely unknown.

## Patients and Methods

### Study Design

We examined four previously described polymorphisms within the IFNAR-1 promoter (27, 30, 31), from now on referred to as interferon-α receptor promoter polymorphisms (IFNARPPs). They include three single-nucleotide polymorphisms (SNPs) at positions −568G/C, −408C/T, and −3C/T and a variable number tandem repeat of the binucleotide GT, −77VNTR(GT)n.

We used 49 (15 WGS and 34 WXS) TCGA “liver hepatocellular carcinoma (LIHC)” samples (project ID 10464). We filtered all the available (up to March 2016) HCC samples with positive HBV surface antigen. We performed genotyping of the four IFNARPPs, using the pipeline described below. We compared the frequencies of the revealed genotypes with those of a group of 92 chronically infected HBeAg-negative IC as defined in our previous study (32) as they present very low risk of cirrhosis and HCC (8–10). Briefly, IC state was defined for patients with persistently normal ALT values under strict follow-up and maximum HBV DNA levels ≤20,000 IU/mL. None of these inactive carriers had cirrhosis (33).

To assess the impact of the −77VNTR and the −3SNP on the expression profiles in HBV-associated hepatocellular carcinoma, we used tumor RNAseq data available for 10 TCGA HCC genotyped samples after classifying them according to their genotype (for −77VNTR: ≤8/≤8 vs. ≤8/>8 or >8/>8(GT) repeats, and for −3SNP: CC vs. CT or TT) (Table 1).

**Table 1:** RNAseq samples analyzed in this study.

Sample	ID	−77VNTR (GTn)	−3SNP (C/T)	Mapped reads (N)	Reads fraction
A	A4NL	≤8/>8	CT	52,242,268	0.93
B	A39Y	≤8/>8	CT	74,221,158	0.95
C	AAV6	≤8/>8	CT	57,894,784	0.92
D	A7ME	>8/>8	CC	63,054,738	0.93
E	A9CZ	>8/>8	CC	52,435,977	0.95
F	A1EF	≤8/≤8	CT	35,808,361	0.94
G	A1EK	≤8/≤8	CT	49,188,130	0.90
H	A66X	≤8/≤8	CC	63,083,028	0.93
I	A7PX	≤8/≤8	CC	50,278,517	0.84
J	A115	≤8/≤8	CC	47,977,943	0.95

### Bioinformatics

**Figure 1:**
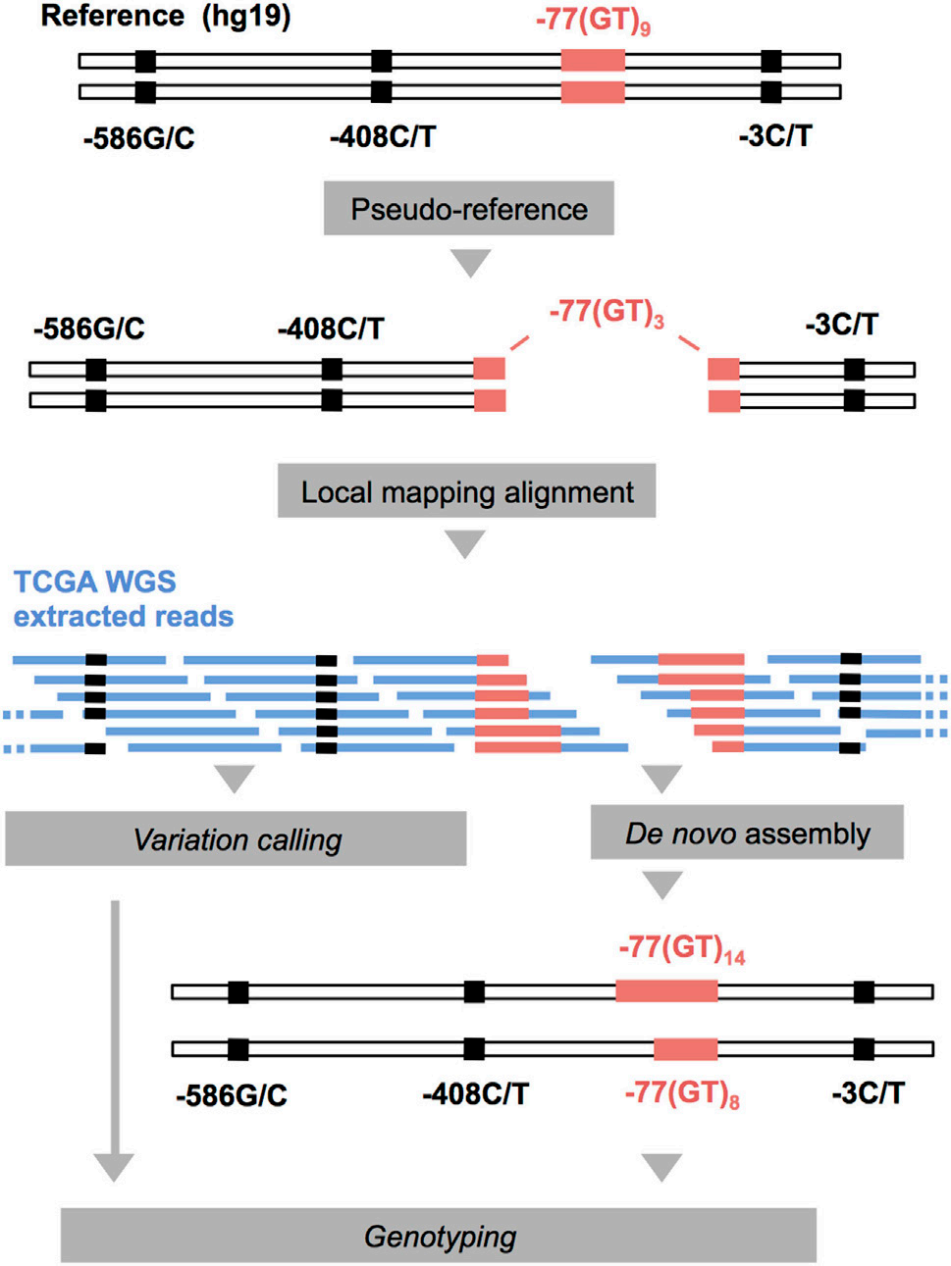
Schematic representation of the computational pipeline applied to whole-genome sequencing (WGS) and WXS The Cancer Genome Atlas (TCGA) data (in blue) for the genotyping of the IFNARPPs. IFNAR1 promoter sequence (genebank: X60459.1) was used to for the construction of two pseudo-chromosomes, on which the reads were aligned. The mapped reads were extracted and used for the *de novo* assembly of the two alleles of each sample. This method allows the assembly of alleles varying substantially in length compared to the reference.

The .bam files were transformed into paired-end .fastq files using Samtools-bam2fq (34) and were locally mapped against two artificial chromosomes using Bowtie2 (35) in—very sensitive local mode (Figure 1). The artificial reference chromosomes were created after splitting the IFNAR1 promoter sequence (genebank: X60459.1) at the −77VNTR, leaving 3 GT repeats at each hanging end, to avoid non-specific mapping of reads due to the low complexity of the microsatellite repeats. We extracted the mapped reads and used them to *de novo* assemble the alleles of IFNAR1 promoter for each TCGA patient, using MIRA (36). We further analyzed the *de novo* assembled contigs using R *DNAstrings* package to count the GT repeats. We used *Samtools mpileup* and *bcftools* (34) to call the variations of the three SNPs from the mapping alignments. The genotyping results were confirmed and quality-controlled by visual inspection of the mapping alignments of 15 random samples, using IGV (37).

We performed the RNAseq analysis using Kallisto (38) to map the reads against the Human Transcriptome reference (v.GRCh38.rel79) and to calculate the transcripts abundances. We analyzed the impact of the IFNARPPs on the transcriptional landscape of the interferon-associated genes using the “Interferome” database (23) on a subset our whole-transcriptome results. We used Sleuth (39) and R-base functions to interpret and visualize the RNA-seq analysis results. We performed Gene Ontology (GO) and KEGG pathway enrichment analysis using the differentially expressed genes (*p* < 0.001).

### Statistical Analysis

We used the *t*-test, the Fisher’s exact test, and the *z*-test to evaluate the association of the demographic and genetic characteristics of the patients with the disease outcome. We used RStudio v0.99.446 for R v3.2.3 programming language for all statistical computing and graphics.

## Results

### RNAseq Differential Expression Design

We tested for differentially expressed transcripts in 10 HCC RNAseq samples after defining their −77VNTR and −3SNP genotypes. We grouped and compared them according to their −77VNTR genotype [samples A–E: >8/≤8 or >8/>8(GT)n vs. F–J: ≤8/≤8(GT)n] and according to their −3SNP genotype (samples A, B, C, F, G: “CT” vs. D, E, H, I, J: “CC”) (Table 1). The quality-control assessment of the RNAseq analysis is summarized in Figure S1 in Supplementary Material.

### The −77 VNTR and −3 SNP Polymorphisms and the IFNAR1 Transcription Profile

IFNAR1 gene generates three different transcripts, but only one of them is translated into the functional receptor protein subunit (transcript “001”: ENST00000270139). Of the two remaining transcripts, one is not translated (transcript “002”: ENST00000493503) and the other (transcript “003”: ENST00000442071) produces a truncated (136 aa long) isoform of the receptor, incorporating only a fibronectin type III domain. We found that −77 VNTR and −3 SNP polymorphisms do not significantly affect the expression levels of the primary transcript “001” and the secondary transcript “002.”

**Figure 2:**
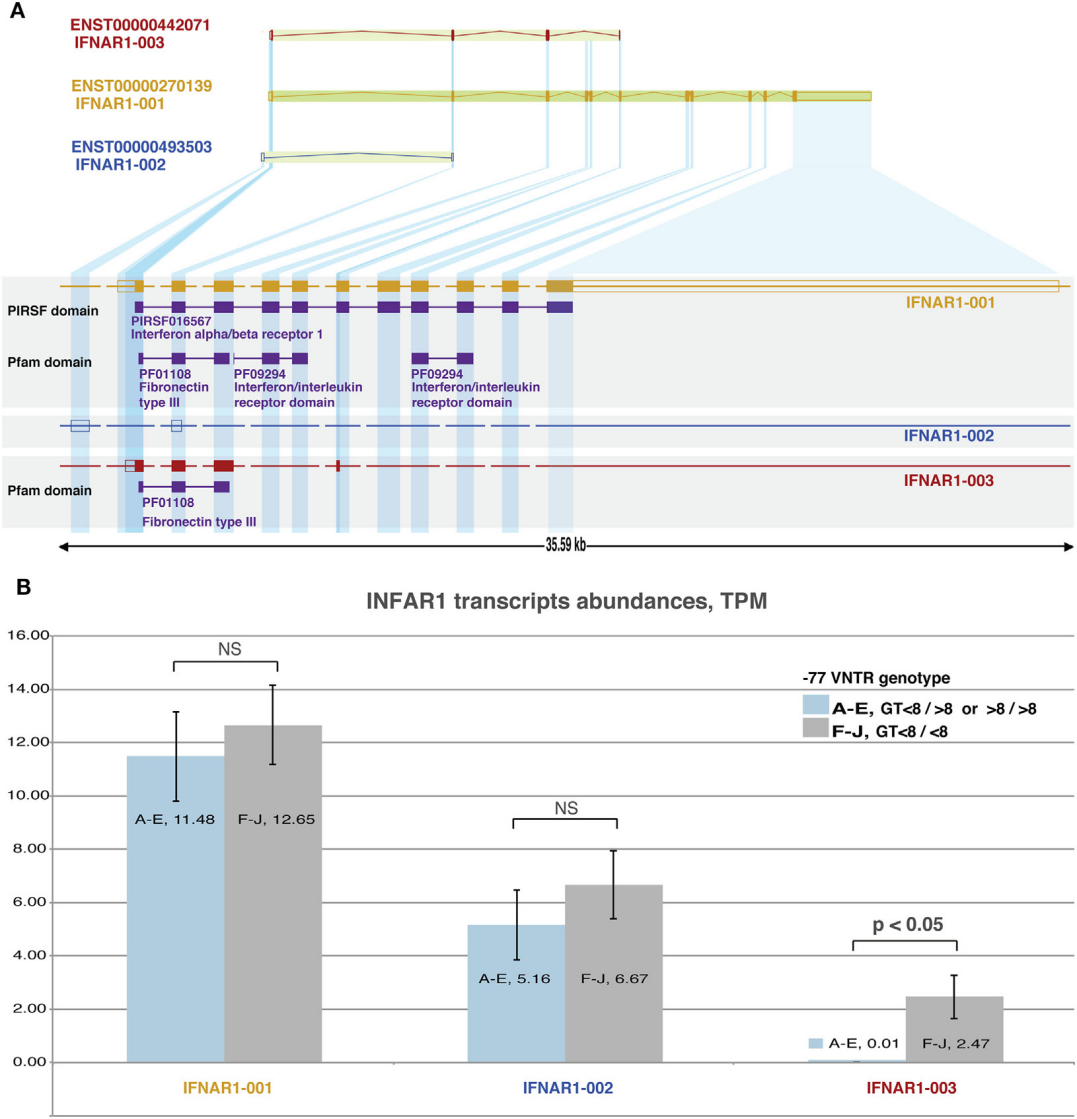
Analysis of IFNAR1 transcripts: **(A)** structure of IFNAR-1 gene. Alternative splicing and transcription initiation sites produce three distinct transcripts that differ in length. Only transcripts 001 and 003 are translated into proteins, which share only the C-terminal part and a fibronectin type III domain. **(B)**: RNAseq-derived expression levels of the three transcripts from 10 hepatocellular carcinoma (HCC) samples grouped based on their −77VNTR(GT)n genotype. The levels of transcripts 001 (responsible for the production of the receptor) and 002 do not differ significantly between the genotypes. Transcript 003 is expressed only in samples with <8/<8 (GT)n repeats in the −77VNTR polymorphism. The genotypes of HCC RNA samples (A–J) are described in Table 1.

Interestingly, the expression of the secondary transcript “003” was detectable only in homozygous patients carrying both alleles with ≤8(GT) repeats, while it was absent in patients carrying at least one allele with >8(GT) repeats (2.47 vs. 0.01 TPM, respectively) (Figure 2). We further tested 10 TCGA normal tissue (liver) RNAseq samples for the expression of this transcript. We found them all negative except from one, which presented only basal expression (0.02 TPM). Thus, transcript “003” was HCC-specific.

### Impact of −77 VNTR and −3 SNP on the HCC Transcriptome and Interferome

**Figure 3:**
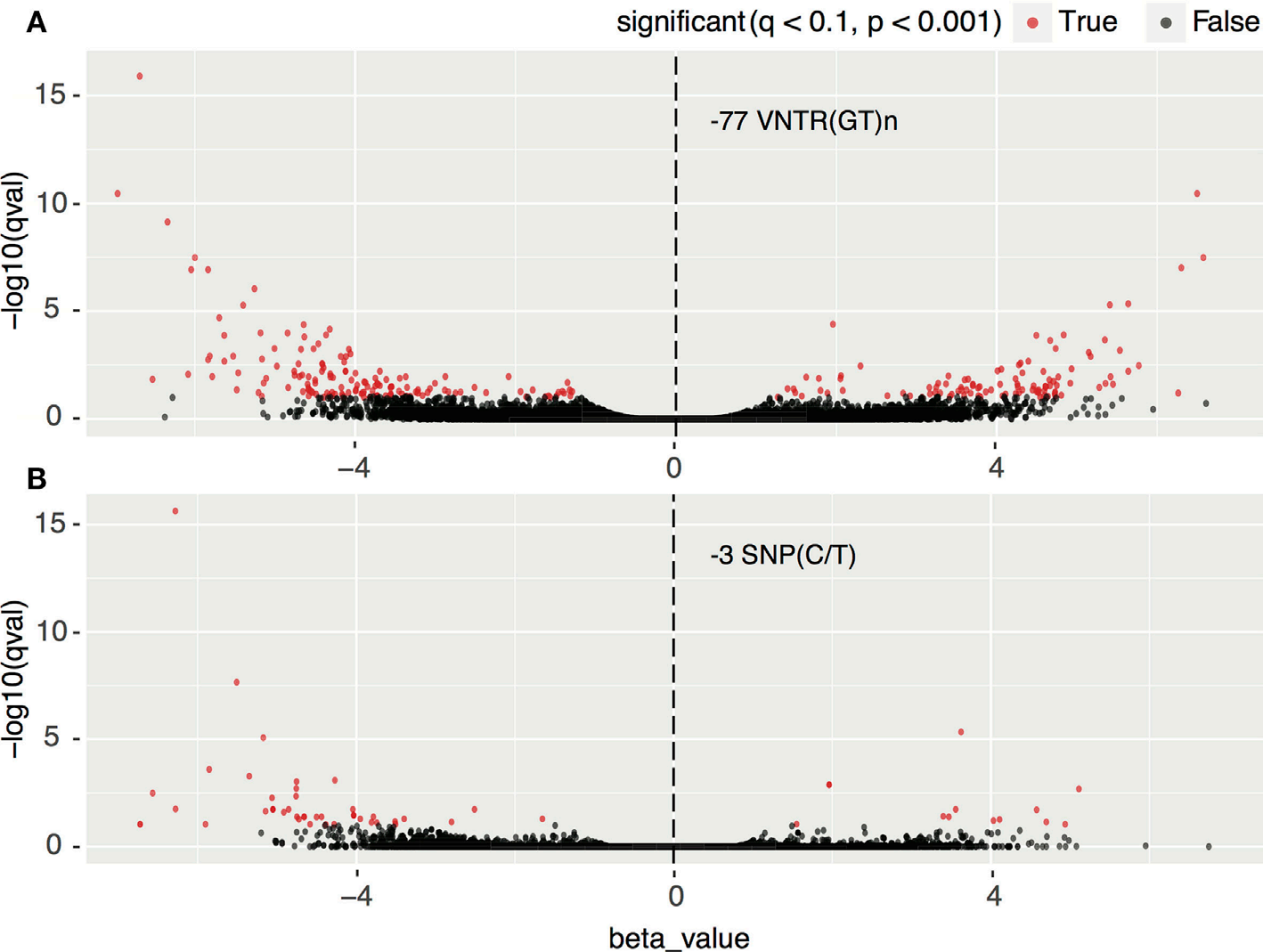
Impact of **(A)** −77 VNTR (GT)n and **(B)** −3SNP C/T on the hepatocellular carcinoma transcriptome. Transcripts with statistically significant change in their expression are in red. Positive and negative normalized beta values correspond to up- or downregulation, respectively. There are approximately four times more significantly differentially expressed transcripts in the −77 VNTR (GT)n compared to the −3SNP C/T genotype group.

We classified 10 HCC TCGA samples according to their genotype, ≤8/≤8 vs. ≤8/>8 or >8/>8(GT) repeats for the −77VNTR, and CC vs. CT or TT for the −3SNP. There were significant changes in the transcription profiles between the groups tested. In detail, for the −77VNTR grouping, there were 246 differentially expressed genes (*p* < 0.001), while for the −3SNP grouping, only 57 genes showed significant changes in their expression levels (*p* < 0.001) (Figure 3).

**Figure 4:**
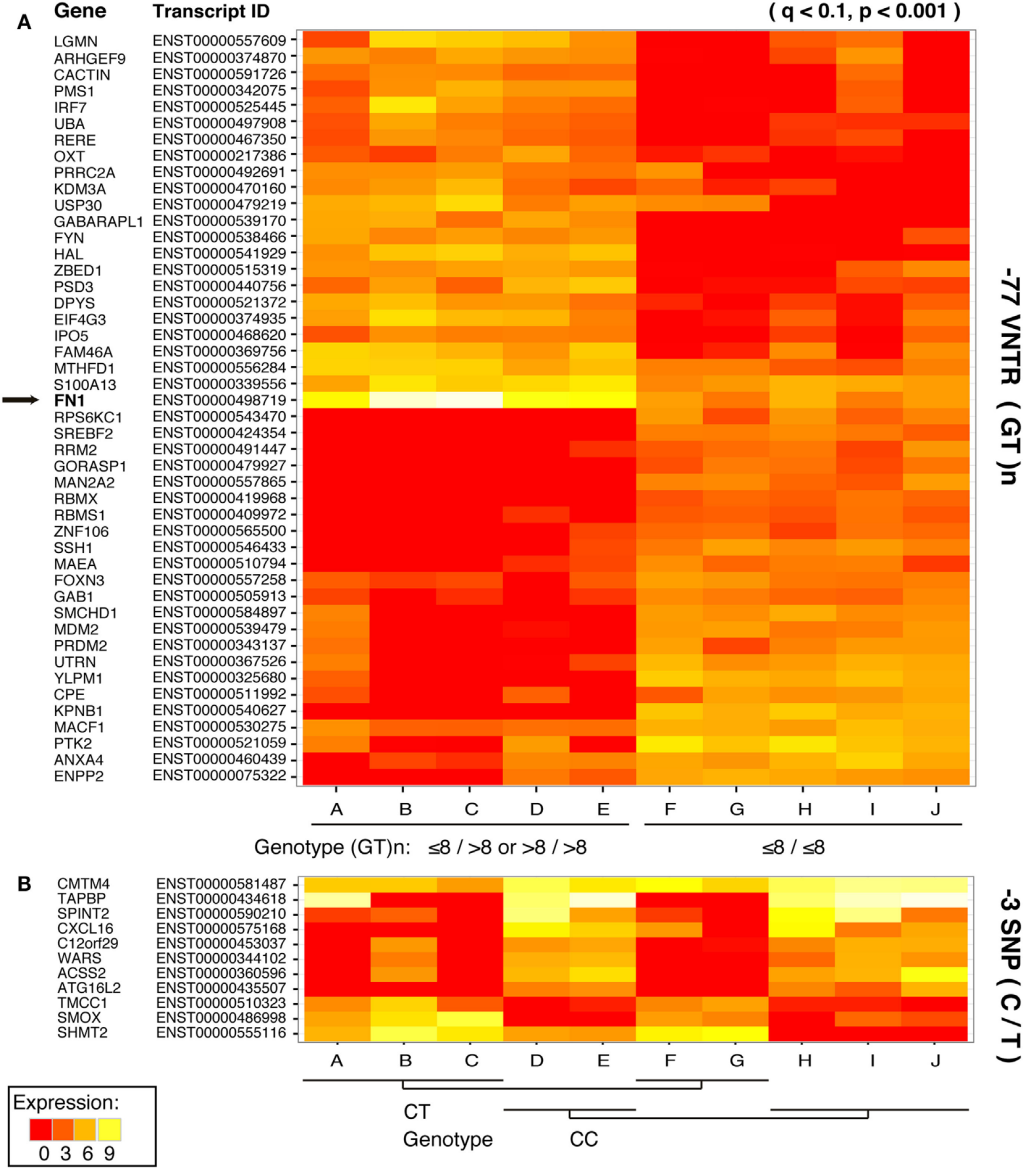
Impact of **(A)** −77 VNTR (GT)n and **(B)** −3SNP C/T on the hepatocellular carcinoma interferome. Only statistically significant differences in the expression levels of the transcripts are presented. Affected transcripts, the respective genes, and the transcripts’ product status are on the left.

Focusing on the interferon-related genes, we created a subset of the transcriptomics results according to transcript IDs found in the *Interferome* database (23). The −77VNTR polymorphism had a fourfold higher impact on the Interferome compared to the −3SNP, as 46 and 11 transcripts, respectively, are either up- or downregulated (*p* < 0.001). Notably, among the most significantly differentially expressed genes was FN-1, which presented a 4.49-fold lower expression in ≤8/≤8(GT)n homozygous patients (Figure 4; Table S2 in Supplementary Material).

### Impact of −77 VNTR on Signaling Pathways

**Figure 5:**
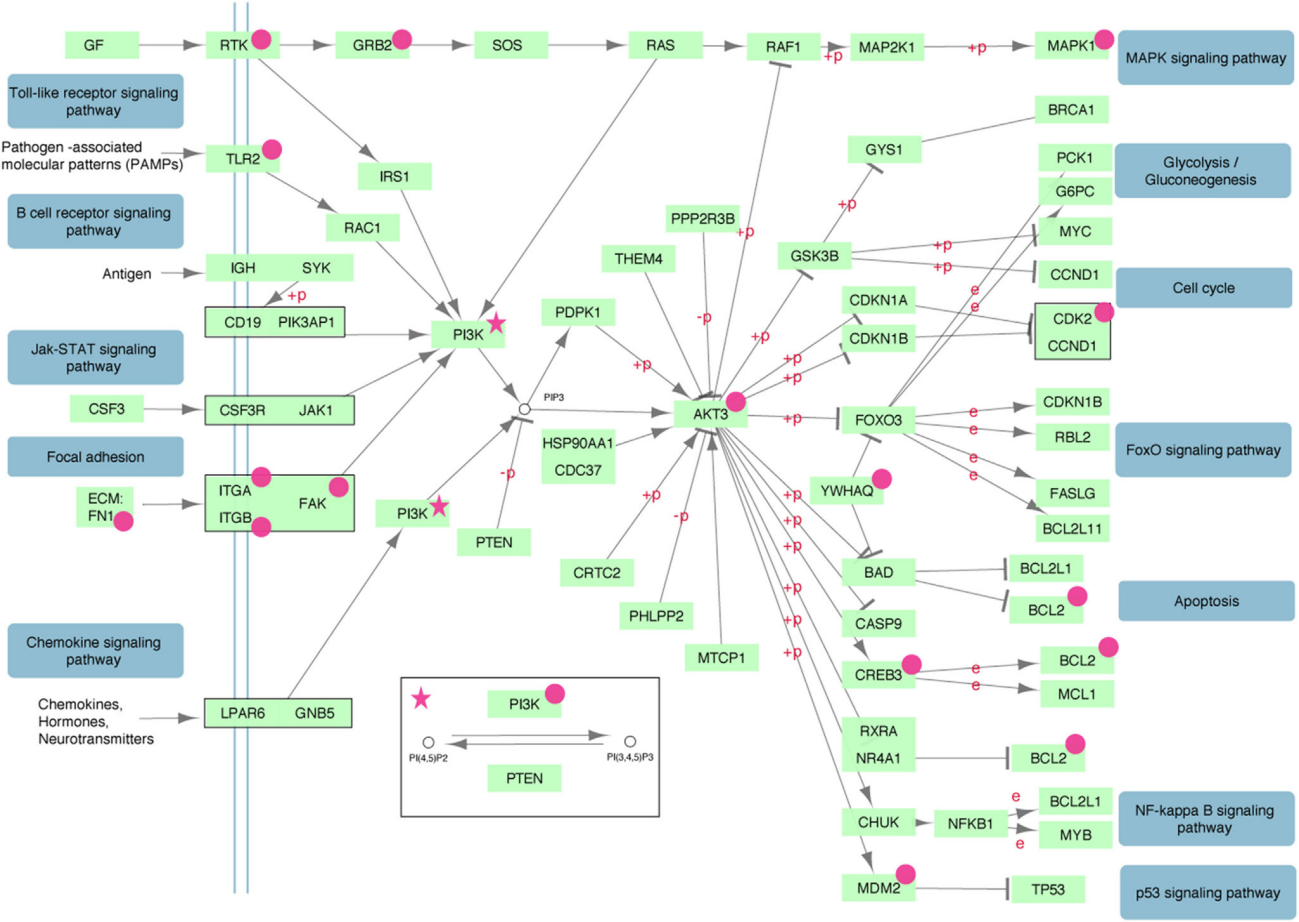
The PI3K–AKT signaling pathway is significantly enriched with differentially expressed genes (magenta). Insulin, ErbB, mTOR, and VEGF signaling sub-pathways triggered by PDK1 have been removed for simplicity.

Performing GO and KEGG pathway enrichment analysis based on the −77VNTR grouping we found six significantly enriched pathways (Table 2). PI3K–AKT signaling pathway was ranked first, with the higher number and proportion of differentially expressed genes involved (14 genes, *p* < 0.05). The super-family “pathways in cancer” was also found significantly enriched with 15 differentially expressed genes, but since PI3K–AKT pathway was the main contributor, the family was excluded from Table 2. Intriguingly, PI3K–AKT signaling pathway can be triggered by FN-1 (Figure 5).

**Table 2:** Pathway enrichment analysis.

Pathway ID: name	Genes count^a^	% of total Diff. Expr. Genes	*p* Value	Fold of enrichment
hsa04151: PI3K–Akt signaling pathway	14	4.13	0.0119	2.15
hsa04510: focal adhesion	13	3.83	<0.001	3.35
hsa05205: proteoglycans in cancer	12	3.54	0.0012	3.19
hsa05203: viral carcinogenesis	10	2.95	0.0142	2.59
hsa04010: MAPK signaling pathway	10	2.95	0.0485	2.08
hsa05166: HTLV-I infection	10	2.95	0.0495	2.08

*^a^A threshold of 10 differentially expressed genes per pathway has been applied*.

### IFNARPPs and HBV-Associated Hepatocellular Carcinoma

We genotyped the four IFNARPPs in 49 HBV-associated HCC samples, 15 WGS and 34 WXS. We were able to confirm the existence of the −568G allele [previously associated with more pathogenic disease states (26, 32)] in 36 out of 49 HCC samples, as the read coverage was occasionally reduced over the −568SNP in the WXS dataset. We compared their distributions of the polymorphisms with those described previously for 92 ICs (32). The demographic characteristics of the 49 HCC and the 92 IC patients are summarized in Table S1 in Supplementary Material. There was no statistically significant difference in frequency of the −568SNP genotypes between the IC and HCC groups (Table 3). Given the previously described linkage between alleles −408C/T and −3C/T (−408C to −3C and −408T to −3T) (27) these were analyzed together. There was no statistically significant difference in the prevalence of the −408/−3 SNP polymorphisms in the IC and the HCC groups (Table 3). The −408/−3 TT genotype was not identified in any of the TCGA samples tested. Notably, the number of GT repeats at the −77VNTR of IFNAR-1 promoter was associated with the disease status. Heterozygous ≤8/>8 (GT)n patients are less likely to be IC compared to homozygotes carrying both alleles with ≤8 GT repeats (OR = 0.41, 95% CI: 0.19, 0.90). The same trend was observed when >8/>8 and ≤8/>8 (GT)n genotypes were grouped together (OR = 0.40, 95% CI: 0.19, 0.84) (Table 3; Figure S2 in Supplementary Material).

**Table 3:** Genotypes of patient samples in relation to clinical status.

	Total (*N* = 141)	By type of HBV infection		*p* Value	Crude OR of being IC (95% CI)
		IC (*N* = 92)	HCC (*N* = 49)		
**IFNARPPs genotypes**					
SNP −568*, *n* (%)				0.313	
CC	54 (42.2)	41 (44.6)	13 (36.1)		1
CG	63 (49.2)	42 (45.7)	21 (58.3)		1.57 (0.65, 3.90)
GG	11 (8.6)	9 (9.8)	2 (5.6)		0.70 (0.06, 4.07)
SNP −408/−3, *n* (%)				0.094	
CC	62 (44.0)	41 (44.6)	21 (42.9)		1
CT	71 (50.4)	43 (46.7)	28 (57.1)		1.26 (0.59, 2.75)
TT	8 (5.7)	8 (8.7)	0 (0.0)		NA
VNTR −77(GT)n, *n* (%)					
≤8/≤8	63 (44.7)	48 (52.2)	15 (30.6)		1
≤8/>8	63 (44.7)	36 (39.1)	27 (55.1)	*****p*** < 0.05**	**0.41 (0.19, 0.90)**
>8/>8	15 (10.6)	8 (8.7)	7 (14.3)		0.36 (0.11, 1.14)
≤8/≤8	63 (44.7)	48 (52.2)	15 (30.6)		1
≤8/>8 or >8/>8	78 (55.3)	44 (47.8)	34 (69.4)	***p* < 0.05**	**0.40 (0.19, 0.84)**

*Statistically significant associations in bold*.

*IC, inactive carrier; HCC, hepatocellular carcinoma*.

*Genotype available only for 118 samples (92 for IC and 36 for HCC).

## Discussion

Implementing a fully automated computational pipeline, we characterized the genetic profile of the four polymorphisms located in the IFNAR1 promoter region in 49 HBV-associated HCC samples derived from the TCGA project.

Whole-genome sequencing data have enormous size and they are usually available as ready-to-use alignments (.bam files), which are binary and compressed. Variation-calling from bam files can be performed with several routine pipelines, each characterized by different biases toward specific types of SNP and in/del genotyping errors (40). For example, reads that differ significantly in length can be ignored and remain unmapped. This is highly likely in VNTRs with heterozygous genotypes in loci where the two alleles differ substantially in length compared to the reference sequence (e.g., −77VNTR(GT)_5/15_), which in combination with low read coverage and read length (quite common in WGS data) can lead to inaccurate variant calling. Our pipeline solves this problem, by using an artificial reference of two pseudo-chromosomes, derived from the original sequence, segregated exactly on the VNTR locus, leaving only 3(GT)-repeats at each segregation edge. By using these two pseudo-chromosomes, we were able to specifically mine all the reads corresponding to the IFNAR1 promoter region—originally mapped or unmapped—that in turn precisely reconstructed the two alleles of each sample. Our method provides biologically meaningful results as only one (homozygous) or two (heterozygous) contigs were generated for all the samples tested. There were not any non-specific contigs generated, indicating that the mining of the reads was highly specific.

Since only 15 TCGA WGS samples met our selection criteria, we used WXS data as well. Although WXS libraries are optimized for mRNA sequencing, non-coding upstream and downstream sequences are randomly captured during the target enrichment process (41, 42). Thus, we were able to accurately genotype the proximate to the first IFNAR1 exon polymorphisms, with the exemption of −568SNP, which could only be genotyped in 36 out of the 49 HCC TCGA samples. −568SNP is the most distant one relatively to the start of the transcription, and this resulted in reduced read coverage in 11 WXS samples. The limited number of observations in the HCC group did not allow for statistically significant results with regard to −568 SNP and disease status, although a relative difference in the genotypic distributions was observed (Figure S2 in Supplementary Material). Further studies are needed to confirm the significance of this SNP, as it has been previously associated with the development of CHB and the spontaneous recovery after acute HBV infection (27, 32).

Our results suggest that the number of GT repeats in the −77VNTR is associated with the state of the disease, as alleles with more than 8(GT)n are more frequent among HCC samples. (Table 3; Figure S2 in Supplementary Material). The finding that more (GT) repeats are associated with a more severe outcome of the disease is in concordance with our previous observations where alleles carrying ≤8(GT) repeats in the −77VNTR, were associated with the IC phase, whereas >8(GT) repeats were more frequent in HBeAg-negative CHB patients (32). Zhou et al. studying CHB patients and spontaneously resolved cases after acute HBV infection showed that alleles carrying ≤9(GT) repeats were more frequent in CHB patients, while >9(GT) repeats were associated with spontaneous clearance after acute HBV infection (27). Moreover, this result was linked with the rest of the polymorphisms examined in haplotypes, [≤9(GT)n, −568C, −408/−3T] for the CHB patients and >9(GT)n, −568G, −408C, −3C in cases of spontaneous clearance after acute HBV infection. In our previous study, alleles −568C and −408/−3T were associated with the IC phase, whereas −568G and −408/−3C were more frequent in CHB patients. In this study, although genotypes −568CG and −408/−3CT show a trend of higher frequency in the HCC samples, compared to genotypes −568CC and −408/−3CC (Figure S2 in Supplementary Material), they were not associated with disease, as the limited availability of HCC samples and the lack of coverage over −568SNP in WXS samples led to lack of statistical power for these comparisons. Notably, genotype −408/−3TT was totally absent in the HCC dataset. Studies in larger datasets may discern whether alleles −568G and −408/−3T and the homozygous genotypes GG and TT are linked to HCC.

The effective expression of IFNAR-1 is essential for the linkage with IFN and the triggering of the downstream signal pathways (43). Even minor modifications in the receptor structure can impair its normal function and alter its antiviral and antiproliferative properties (44, 45). Moreover, the expression levels of both the receptor subunits IFNAR1 and IFNAR2 are associated with the INF-β treatment outcome in multiple sclerosis patients (46). Hepatitis C patients with 5GT repeats in the −77VNTR (genotypes GT_5/5_/GT_5/14_) have been reported to be better responders to IFN-based therapy (47). On the other hand, experiments based on luciferase reporter plasmids have suggested that the promoter of the IFNAR-1 is not affected by the −568SNP and the −77VNTR (26, 27), while in another study its activity is significantly affected by −3SNP (48). In detail, the promoter activity was reduced for −3T plasmids through reduced binding affinity to HMGB1, a factor that was suggested to bind to the −3 element to regulate the transcription levels of IFNAR1. Similarly, reduced levels of IFNAR1 expression in HBV patients with C > T substitution at the −3 position of the IFNAR1 promoter was reported in the same study (48). In another study, an important role of the −3CT SNP was evident regarding the regulation of the transcription factor High Mobility Group B protein 1 (HMGB-1); the C > T transition was shown to reduce the binding affinity of HMGB-1 to the IFNAR-1 promoter sequence, thus lead to reduced expression of IFNAR-1 (48).

In this study, we conducted a thorough RNAseq differential expression analysis on HCC samples grouped according to their −77VNTR and −3SNP genotype. In agreement with our previous findings, where a significant role of the −77VNTR genotypes had arisen with respect to the clinical course of HBV infection, here we report a more significant involvement of −77VNTR in the modification of the HCC transcriptome and interferome, as more genes were found to be significantly differentially expressed compared to those affected by the −3SNP. We also shed light on the controversial question about the impact of these promoter polymorphisms on the expression of IFNAR-1 gene. The expression levels of the receptor are associated with the clinical outcome of chronically HBV-infected patients (49). We conclude that the expression of the major IFNAR-1 transcript, which is responsible for the production of the functional receptor, is not significantly affected by these polymorphisms (Figure 2).

At the same time, a secondary IFNAR1 transcript, which is translated into a truncated form of the receptor, is only produced at detectable levels in samples with ≤8/≤8 (GT)n genotype in the −77VNTR, independently from the −3SNP genotype. This transcript is not detectable in normal liver tissue samples, thus appears to be HCC specific. Secondary IFNAR1 transcripts have been reported in tumor cell lines (50) and here, for the first time, we report that −77VNTR genotype is crucial for their production, which apparently leads to the transcriptional remodeling of IFNAR1 gene.

Grouping the samples according to their −77VNTR genotype revealed that one of the most differentially expressed genes was the extracellular matrix FN-1. Patients carrying at least one −77VNTR >8(GT) allele, presented a strong upregulation of the FN-1 gene, while FN-1 was significantly downregulated in ≤8/≤8(GT)-repeats carrying patients, a phenomenon coinciding with the expression of the secondary IFNAR-1 transcript, which implies its potentially protective role against the development of HCC. This transcript comprises only a fibronectin III domain; FN fragments and modules can inhibit FN-matrix assembly by competing for FN-assembly sites, which could act as a feedback system to regulate FN levels on the cell surface (51). Specifically, the first type III domain of the FN molecule is important for the matrix assembly (52, 53), while even small fragments derived from this module regulate FN polymerization, inducing it at moderate concentrations but inhibiting it at high concentrations (54, 55). FN-1 is involved in HCC as it can be upregulated by HBxAg in an NFkB-dependent way (14), while is generally over-expressed in several cancers [reviewed in Ref. (56)]. NFkB was not found differentially expressed in our dataset (*p* = 0.16) but is linked to PI3K-AKT, which was the most significantly enriched signaling pathway, down to the MDM2 proto-oncogene and the apoptosis regulator Bcl-2 (Figure 5). FN-1 can trigger this pathway and transduce multiple intracellular signals that control cell cycle. Our data suggest that apart from NFkB, complementary mechanisms, partially controlled by FN-1 and involved in cell cycle regulation might exist.

This study is based on data mining and *in silico* analyses, thus our findings here generate a strong hypothesis about links between the expression of the truncated IFNAR-1 transcript either with the PI3K–AKT signaling pathway and/or with the HCC development. To test this hypothesis, further wet-lab studies will be needed involving knock-down of the transcript, transfection with a plasmid that will produce this transcript and quantitative analysis of the respective transcripts and proteins would confirm these observations and would shed light in these complex regulatory networks.

In conclusion, our results suggest very minimal (if any) involvement of the IFNAR-1 promoter polymorphisms in the expression levels of the IFNAR-1 major transcript but at the same time raise a potential and intriguing role for the −77VNTR regarding the regulation of downstream genes. Our study shows that the majority of modifications of the *Interferome* coincided with the production of the truncated IFNAR-1 transcript. Thus, further study of this truncated transcript could clarify the mechanistic features of the combined antiviral and anticancer roles of IFNAR-1.

## Author Contributions

TK designed and conducted the analyses, evaluated the results, and wrote the manuscript. GP, DP, AH, JM, UG, PK, and GM wrote and revised the manuscript.

## Conflict of Interest Statement

The authors declare that the research was conducted in the absence of any commercial or financial relationships that could be construed as a potential conflict of interest.
