# Supplementary material for: Impact of Interferon-α Receptor-1 Promoter Polymorphisms on the Transcriptome of the Hepatitis B Virus-Associated Hepatocellular Carcinoma
Source: Front Immunol. 2018 Apr 16;9:777. doi: 10.3389/fimmu.2018.00777 (PMC5911724; doi:10.3389/fimmu.2018.00777)
Supplement: Supplementary file 1 [file table_1.PDF]

Supplementary Table 1

Demographic characteristics of the patients

	Total (N=141)	By type of HBV infection	
		**IC a (N=92)**	**HCC b (N=49)**
Gender, n(%)			
Female	54(38.3)	38(41.3)	16(32.7)
Male	87(61.7)	54(58.7)	33(67.3)
Age			
mean, years (SD)		44.2 (14.4)	61.8(14.4)
