# Supplementary material for: Impact of Interferon-α Receptor-1 Promoter Polymorphisms on the Transcriptome of the Hepatitis B Virus-Associated Hepatocellular Carcinoma
Source: Front Immunol. 2018 Apr 16;9:777. doi: 10.3389/fimmu.2018.00777 (PMC5911724; doi:10.3389/fimmu.2018.00777)
Supplement: Supplementary file 2 [file table_2.PDF]

Supplementary Table 2

Interferon associated, differentially expressed genes, and their relation with cancer.

**Gene Name**	**Description**	**Reference**
Protein tyrosine kinase 2 (PTK2 or PYK2) or Focal Adhesion Kinase (FAK)	Involved in cellular adhesion and spreading processes. Associated with HCC and HBV infection	Okamoto, H., et al., Hepatology, 2003. **38**(5): p. 1242-9.
FAM46A (alternative names: HBV X-transactivated gene 11 protein, C6orf37, XTP11)	Trans-activated by HBV protein X, associated with retinal dysfunction	Lagali, P.S., et al., Biochem Biophys Res Commun, 2002. **293**(1): p. 356-65.
Oxytocin (OXT)	Growth regulator, in choriocarcinoma cells, on normal and neoplastic trophoblasts and also on a Kaposi sarcoma-derived cell line	Cassoni, P., et al., Endocrinology, 2001. **142**(3): p. 1130-6.<br>Cassoni, P., et al., Cancer Res, 2002. **62**(8): p. 2406-13.
Eukaryotic translation initiation factor 4 gamma 3 (EIF4G3 / eIF4GII)	Down-regulated by miR-520c-3p can lead to inhibition of cell proliferation in B Cell Lymphoma	Mazan-Mamczarz, K., et al., PLoS Genet, 2014. **10**(1): p. e1004105
Calcium-binding protein A13 (S100A13)	Up- regulated in high-grade vascularized gliomas	Landriscina, M., et al., J Neurooncol, 2006. **80**(3): p. 251-9.
Forkhead box protein N3 (FOXN3), also known as CHES1	Reduced in many types of cancers. Short hairpin RNA-mediated depletion of CHES1 increases tumor cell proliferation	Huot, G., et al.,. Mol Biol Cell, 2014. **25**(5): p. 554-65.
Acetyl-CoA synthetase 2 (ACSS2)	Contributes to cancer cell growth under low-oxygen and lipid-depleted conditions	Schug, Z.T., et al., Cancer Cell, 2015. **27**(1): p. 57-71.
