# Supplementary material for: Impact of Interferon-α Receptor-1 Promoter Polymorphisms on the Transcriptome of the Hepatitis B Virus-Associated Hepatocellular Carcinoma
Source: Front Immunol. 2018 Apr 16;9:777. doi: 10.3389/fimmu.2018.00777 (PMC5911724; doi:10.3389/fimmu.2018.00777)
Supplement: Supplementary file 3 [file image_1.PDF]

Supplementary Figure 1:

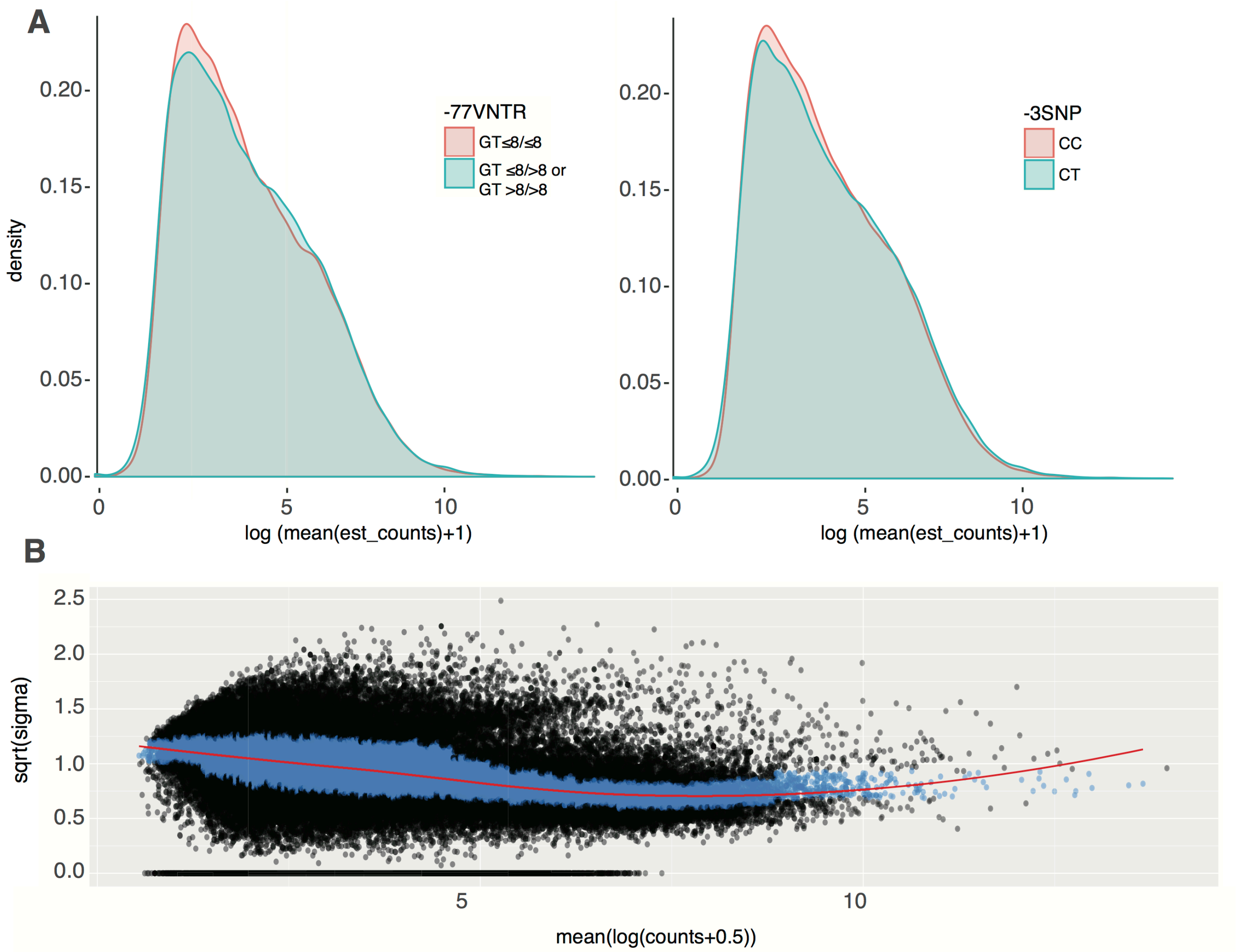

Supplementary Figure 1: A. Distribution (density plots) of reads abundances (estimated counts) between the sample groups tested each time: VNTR -77 grouping is on the left and SNP -3 grouping is on the right. B. Abundance of transcripts vs. the square root of standard deviation (mean variance plot). Blue dots represent the interquartile range. The red line represents the fit of the data to the *Sleuth* shrinkage model.
