# Supplementary material for: Impact of Interferon-α Receptor-1 Promoter Polymorphisms on the Transcriptome of the Hepatitis B Virus-Associated Hepatocellular Carcinoma
Source: Front Immunol. 2018 Apr 16;9:777. doi: 10.3389/fimmu.2018.00777 (PMC5911724; doi:10.3389/fimmu.2018.00777)
Supplement: Supplementary file 4 [file image_2.PDF]

Supplementary Figure 2:

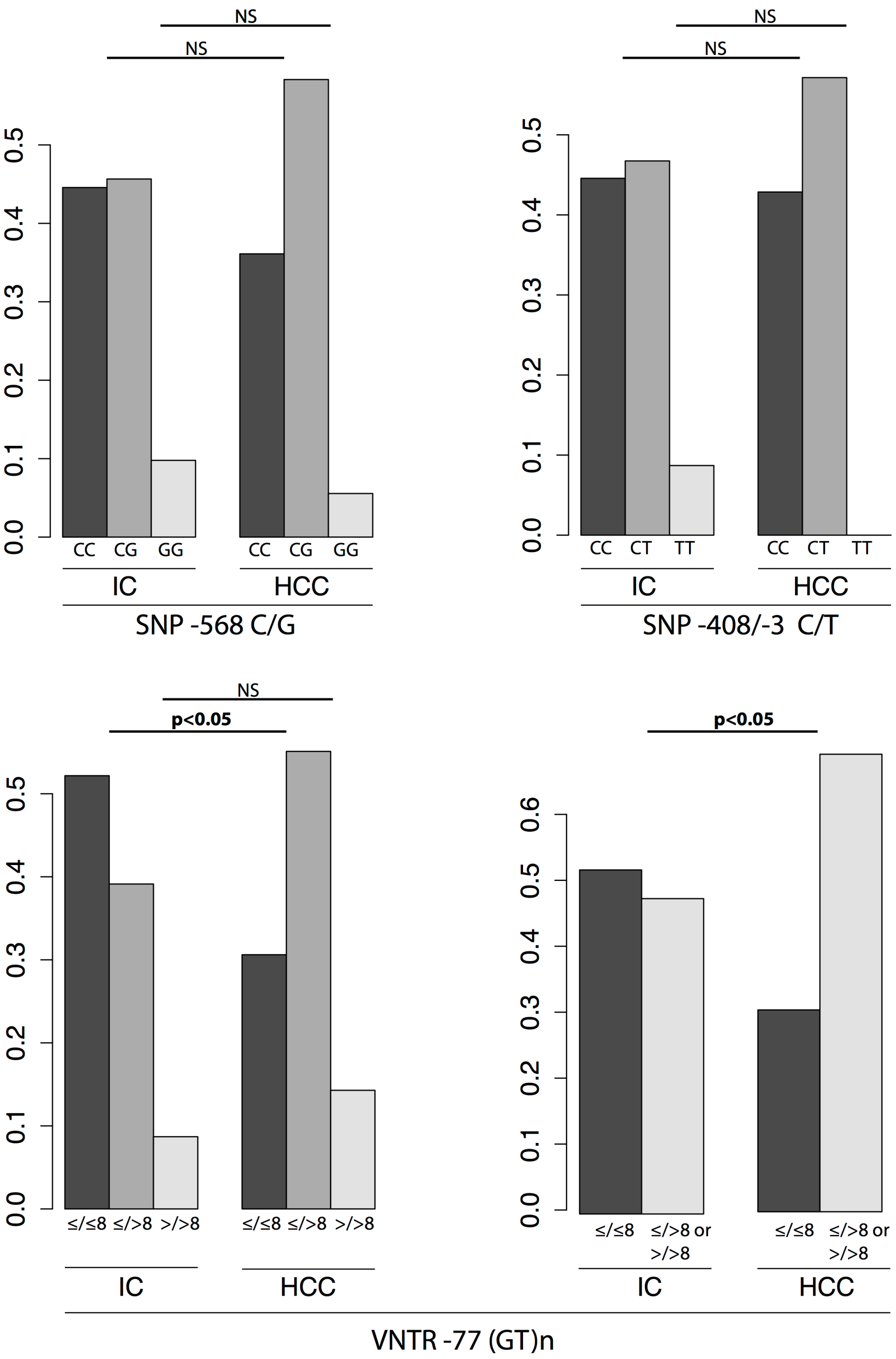

Supplementary Figure 2: Comparison of HBeAg(-) Inactive Carriers (IC) and Hepatocellular Carcinoma (HCC) samples. Genotype prevalence of SNP -568, SNP -408/-3 and VNTR -77. VNTR -77 was examined with or without grouping of >8/>8 and ≤8/>8 (GT)n patients. Statistically significant associations are in bold.
